# Dissecting the tumor micro-environment in triple negative breast cancer identifies a mutually exclusive expression pattern of the immune co-inhibitory molecules B7-H4 and PD-L1

**DOI:** 10.1186/2051-1426-3-S2-O17

**Published:** 2015-11-04

**Authors:** Donald R Shaffer, Virna Cortez-Retamozo, Kumiko Nagashima, Tong Zi, ChengYi J Shu, Igor Feldman, Veronique Neumeister, Jeff Smith, Mohammad Zafari, Rebecca Larson, Matthew Silver, Robert Mabry, Michael Briskin, Tanya Novobrantseva, David Rimm, Sriram Sathyanarayanan

**Affiliations:** 1 Jounce Therapeutics, Cambridge, MA, USA; 2 Department of Pathology, Yale University, New Haven, CT, USA; 3 Cell Signaling Technologies, Danvers, MA, USA; 4 Yale University School of Medicine, New Haven, CT, USA

## 

B7-H4 is a member of the B7 family of co-regulatory receptors. It is believed to negatively regulate T cell function and has been associated with poor prognosis in renal cell and ovarian cancers. We performed an unbiased analysis of TCGA gene expression data and identified triple negative breast cancer (TNBC) as having the greatest absolute B7-H4 mRNA level of all tumors analyzed. Recent clinical studies with anti-PD-1 or PD-L1 therapies have reported promising activity in TNBC leading us to investigate check point molecule expression (PD-1, PD-L1 and B7-H4) as well as expression of immune cell infiltration (CD8, Fox P3) in archival samples from a cohort of 96 TNBC patients collected at Yale University.

We developed a specific and sensitive immunohistochemistry (IHC) assay for evaluating B7-H4 protein and used an immunofluorescence-based multiplex IHC for assessing combinations of checkpoint molecules in the TNBC samples. The majority of tumors had detectable B7-H4 expression, whereas PD-L1 expression was restricted to a subset of TNBC patients (~20% having >5% PD-L1 positive cells). Multiplex IHC and flow cytometry studies showed that the majority of B7-H4 expression was restricted to the tumor epithelial cells, while the CD45+ immune cells were negative for B7-H4 expression. Interestingly, a majority of the B7-H4 high tumors were negative or showed scant PD-L1 staining. In addition, cells that are B7-H4 positive are negative for PD-L1 staining, suggesting that B7-H4 and PD-L1 checkpoint proteins may act in a mutually exclusive manner. B7-H4 expression was not associated with overall survival, disease stage, nodal status, or other clinical characteristics. In contrast, PD-L1, PD-1, and CD8 expression all conferred a significant survival advantage in TNBC, thus highlighting the importance of the immune response in this disease.

Upon further investigation, and contrary to published literature, we were unable to show a definitive immunosuppressive role of B7-H4. However B7-H4 over-expression in CT-26 syngeneic in vivo model accelerated tumor growth. The unique expression pattern of B7-H4 on TNBC suggests an opportunity for targeted approaches with possible immunomodulatory activity. Additional work is needed to further clarify the immunological mechanisms of B7-H4, but we believe its unique expression pattern makes B7-H4 an attractive target for the treatment of TNBC.

